# Genetic Alterations in Pesticide Exposed Bolivian Farmers: An evaluation by analysis of chromosomal aberrations and the comet assay

**Published:** 2007-11-12

**Authors:** Erik Jørs, Ana Rosa Gonzáles, Maria Eugenia Ascarrunz, Noemi Tirado, Catharina Takahashi, Erika Lafuente, Raquel A Dos Santos, Natalia Bailon, Rafael Cervantes, Huici O, Jesper Bælum, Flemming Lander.

**Affiliations:** 1 Department of Occupational and Environmental Medicine, Odense University Hospital, Denmark; 2 Unidad de Genética Toxicológica. Instituto de Genética, Facultad de Medicina, Universidad Mayor de San Andrés, La Paz, Bolivia; 3 Proyecto PLAGBOL, La Paz, Bolivia; 4 Department of Occupational and Environmental Medicine, Skive Hospital, Denmark

**Keywords:** pesticides, agriculture, chromosomal aberrations, comet assay, Bolivia

## Abstract

**Background:**

Pesticides are of concern in Bolivia because of increasing use. Frequent intoxications have been demonstrated due to use of very toxic pesticides, insufficient control of distribution and sale and little knowledge among farmers of protective measures and hygienic procedures.

**Method:**

Questionnaires were applied and blood tests taken from 81 volunteers from La Paz County, of whom 48 were pesticide exposed farmers and 33 non-exposed controls. Sixty males and 21 females participated with a mean age of 37.3 years (range 17–76). Data of exposure and possible genetic damage were collected and evaluated by well known statistical methods, controlling for relevant confounders. To measure genetic damage chromosomal aberrations and the comet assay analysis were performed.

**Results:**

Pesticide exposed farmers had a higher degree of genetic damage compared to the control group. The number of chromosomal aberrations increased with the intensity of pesticide exposure. Females had a lower number of chromosomal aberrations than males, and people living at altitudes above 2500 metres seemed to exhibit more DNA damage measured by the comet assay.

**Conclusions:**

Bolivian farmers showed signs of genotoxic damage, probably related to exposure to pesticides. Due to the potentially negative long term health effects of genetic damage on reproduction and the development of cancer, preventive measures are recommended. Effective control with imports and sales, banning of the most toxic pesticides, education and information are possible measures, which could help preventing the negative effects of pesticides on human health and the environment.

## Background

During the last decades the use of pesticides has increased steadily in developing countries in an effort to increase food production and control vector-borne diseases. Unfortunately this has resulted in some negative side effects on human health and the environment. Many people are potentially at risk of becoming intoxicated when using pesticides in agriculture, in health vector programmes, and at home. Moreover people trying to commit suicide and consumers eating pesticide contaminated foods are at risk, due to the easy accessibility of pesticides and the high levels of pesticide residues found in e.g. vegetables on the market (Maroni et al. 2006; Hamilton et al. 2004; Konradsen et al. 2003).

The pesticides used are among the most toxic ones and there is often insufficient control with imports and sales. The farmers who use pesticides have only little or no access to information about proper use or the precautions needed when handling pesticides. Therefore, they often do not use even the simplest hygienic and protective measures (Maroni et al. 2006; Jørs et al. 2006; Gomes et al. 1999).

The most obvious health problem is the large number of acute and often fatal intoxications seen. Chronic health problems such as respiratory diseases, dermatitis, and neurological disorders are also reported (McCauley et al. 2006; Maroni et al. 2006). Increased genetic damage has been shown among pesticide users (Sailaja et al. 2006; Castillo-Cadena et al. 2006; Bolognesi, 2003; Lander et al. 2000), and is of concern because it might lead to miscarriages, birth defects, and cancer (Jaga et al. 2005; Hagmar et al. 2004; Sasaki et al. 2000; Sigurdson et al. 2005).

In this study the possible genotoxic effects of pesticides among exposed small-scale farmers are evaluated as part of the PLAGBOL project whose primary goal is the prevention of negative health effects of pesticides by intervention directed mainly towards farmers and health personnel in Bolivia (www.plagbol.org.bo).

## Materials and Methods

### The study population

The study was carried out in 2004 in La Paz County in Bolivia, a mountainous area with altitude variations from 1000 to 4000 meters above sea level and climatic variations from temperate to subtropical. The study involved volunteers from four communities, two in the highlands above 2500 m and two in the valleys from 1000–2500 m above sea level. The usual method when applying pesticides was knapsack sprayers and the farmers exposed themselves to a mixture of pesticides, including the very toxic methyl parathion and metamidophos, see Table 1. The spraying season covered the whole year but was especially intensive in the hot and rainy period from October to April, when spraying might take place several times a week (Jørs, 2004).

Initially the study included one hundred individuals, but due to problems with blood analysis in 19 of the samples, the study ended up with 81 persons. Of these 48 were pesticide using farmers, who mainly grew tomatoes, flowers and vegetables, and 33 unexposed controls consisting of health care workers, teachers and ecological farmers from the same area. The study included interviews and blood tests. For background data see Table 2. Exclusions criterias were presence of serious chronic disease, recent exposure to cytotoxic medicine or recent exposure to x-rays.

### Laboratory methods

Blood samples were drawn from a cubital vein into heparinized vacutainers, cooled down, and within 6 hours processed at the Genetic Institute at the University Mayor de San Andrés’ in La Paz, where slides for analyses of chromosomal aberrations and DNA damage by the comet assay were prepared. Analysis of chromosomal aberrations was done in La Paz, and the comet assay analysis at the ‘Universidad Tecnica Particular de Loja’ in Quito, Ecuador. The samples were coded at the time of preparation and subjects to blinded analysis.

### Chromosomal aberrations assay

0.25 ml of venous blood was taken from each subject using heparinized vacutainer tubes. Whole blood was cultured in 7 ml of RPMI-1640 medium supplemented with 20% fetal bovine serum, 10000 IU/ml penicillin G sodium, 10000 ug/ml streptomycin sulfate, 25 ug/ml amphotericin B in 0,85% saline and 0,15 ml phytohaemaglutinin (Gibco). Each culture was incubated at 37 °C for 72 hours. Metaphases were obtained by adding 0.25 ml colchicine 0,001% to the cultures 2 hours before harvesting. The lymphocyte cells were collected by centrifugation at 800 rpm and suspended in a preheated hypotonic solution of 0,075 M KCl for 15 min at 37° and fixed 3 times in acetic acid: methanol (1:3, v/v). Cells were suspended in 0.3 to 0.5 ml of fixative solution and dropped onto the slides; the slides were stained with 2% Giemsa solution for 5 min. in Sorensen buffer pH 6.8 and air dried. The slides were analyzed at 1000x magnification using a light microscope, and 100 meta-phase cells were screened per individual. Cells with 46 chromosomes were scored for chromosome and chromatide gaps and breaks.

### Comet assay

Venous blood samples were collected using heparinized vacutainer tubes. The samples were coded and transported on ice to the laboratory and processed on the same day. Three slides were prepared per subject. The slides were precoated with 1.5% normal melting point agars (40–42 °C) and allowed to gel at 4 °C for 10 min. An aliquot of 20 μl of whole blood was then added to 0.7% of 110 μl of low melting point agars (37 °C) and allowed to solidify at 4 °C for 10 min, with the cover slips in place. To lyse cellular and nuclear membranes of the embedded cells and to permit DNA unfolding in alkaline conditions, the cover was removed and the slides were immersed in ice-cold freshly prepared lyses solution (2.5 M NaCl, 100 mM Na2EDTA, 10 mM Tris-HCl, pH 10, 1% Triton X-100 and 10% DMSO) for 1 h at 4 °C. The slides were then placed in alkaline buffer (1 mM Na2EDTA, 300 mM NaOH, pH 13) for 20 min to allow unwinding of the DNA. Electrophoresis was conducted for 20 min at 25 V (0.66 V/cm) adjusted to 300 mA by raising or lowering the buffer level in the tank. Slides were then drained, placed on a tray and washed slowly with three changes of neutralizing buffer (0.4 M Tris-HCl, pH 7.5) for 5 min each. DNA was precipitated and slides were dehydrated in absolute ethanol for 10 min and left to dry at room temperature. The whole procedure was carried out in dimmed light to minimize artifact DNA damage. Slides were stained with ethidium bromide (20 ug/ml).

The lymphocytes were examined with a fluorescent microscope using an automated system with a camera connected to a computer using the CASP package for analysis (http://free.of.pl/c/casp/). From the parameters delivered by the CASP comet assay analysis, we present the area of the comet tail, % of DNA in the comet tail, length of the comet tail, and comet tail moment (% of DNA in the tail × length of the tail), all measures of DNA migration from the cell indicating DNA-damage.

### Data analysis and statistics

Differences between the exposed farmers and the control group were tested using a simple non parametric rank sum analysis. General linear model methods (GLM) were used to include confounding factors. For the chromosome aberrations a Poisson distribution was chosen, while the different variables of the Comet assay were logarithmically transformed. The exposed group of farmers was divided into three categories, those having sprayed two or more times during the previous month, those having sprayed once and those not having sprayed within the last month. Smoking habits, sex, age (≤35 and >35) and altitude of living above sea level (high >2500 m or low <2500 m) were tested for correlation with exposure. Only altitude and sex were correlated with the exposure status and therefore included in the final statistical models. All analyses were made on the STATA software package version 8 (StataCorp, College Station, Texas).

### Ethical considerations

The participants were informed about the health dangers a blood test might pose and signed an informed consent before they were included in the study. The study is in compliance with the Helsinki Declaration and was approved by the Medical Ethical Committee in Bolivia.

## Results

The total number of chromosome aberrations was 8.38 ± 9.67 (mean, SD) among exposed farmers and 2.53 ± 4.79 among controls (p < 0.001). The average number of chromosome and chromatide breaks and gaps seen in different exposure groups and in groups with different living altitude is shown in Table 3. A dose related increase in chromosome aberrations was seen, although not significant for chromatide gaps (Table 4). Sex and altitude were included as possible confounders in the analysis, and sex was found to be an independent risk factor.

The mean level of DNA damage in lymphocytes compared among different exposure groups and in groups with different living altitude is shown in Table 5. The exposed farmers had significantly higher DNA damage in all the comet assay parameters than the controls, but no clear difference was seen between the high and low exposure groups of farmers (Table 5 and 6). Living altitude seemed to be an independent risk factor, while sex was not (Table 6).

Correlations between the variables in the comet assay and those of the chromosome aberrations subtypes were low with coefficiences R around 0.20.

## Discussion

This study shows that pesticide exposed farmers, when compared to non-exposed controls, have a higher degree of genetic damage in their peripheral lymphocytes.

A dose response correlation with the number of recent application and chromosome aberrations is in accordance with an acute effect of pesticide application, and this is also seen in studies from both developing and industrialized countries. The percentage of total chromosomal aberrations of 8% among exposed farmers found in this study is comparable to other studies with findings of chromosomal aberrations between <1% to >20% among exposed groups (Sailaja et al. 2006; Paz-y-Miño et al. 2002; Lander et al. 2000).

Likewise the comet assay shows more DNA damage in the lymphocytes of farmers spraying pesticides than in the control group, which is also seen in other studies (Castillo-Cadena et al. 2006; Garaj-Vrhovac et al. 2000; Lebailly et al. 1998). This damage is probably due to pesticides inducing oxidative stress in the cells, leading to strand breaks of DNA, alkali-labile sites and transient repair sites (Møller, 2006a). The lack of correlation with the level of recent exposure suggests a longer lasting effect on the comet assay parameters than that on the chromosome aberrations.

Both pesticide exposed farmers and controls living at high altitudes show significantly more DNA-damage than those living at lower altitude. This finding could be due to higher exposure to UV-radiation at the altitude or more hours in the sunlight, a finding that might parallel an earlier finding were latitude seemed to be correlated to the degree of DNA damage (Møller, 2006b).

We did not find any relation with age or smoking habits, while sex was found to influence the number of chromosomal aberrations. Such varying results are also shown in other studies (Bolognesi 2006; Castillo-Cadena et al. 2006; Møller, 2006a–b; Kourakis et al. 1996). The reason why sex influences the number of chromosomal aberrations could be a more hygienic handling and thus less exposure to pesticides among women. In hot climates we have seen that males are often more or less undressed when working, while women keep their clothes on and thus reduce the chance of dermal exposure.

A study found a positive correlation between the length of pesticide exposure and DNA damage, and one showed a decrease in the comet tail length after 8 months of non-exposure in the same group of workers, as an indication of the role of pesticides in producing DNA damage (Bolognesi, 2003; Ramirez and Cuenca, 2002; Garaj-Vrhovac et al. 2000). Lebailly et al. (1998) showed DNA damage in persons after one day of spraying with pesticide mixtures including the herbicide isoproturon as well as the fungicides chlorothalonil and triazoles. While most studies show a difference in genotoxic changes among pesticide exposed and non-exposed individuals, some do not find such differences (Bolognesi, 2006; Piperakis et al. 2006). These different findings might be due to varying circumstances of exposure.

In Bolivia the subsistence farmers are exposed to pesticides most of the year because the climate allows several harvests per year. They use very toxic pesticides and have little knowledge about safe use, and that is probably one of the reasons why personal protective equipment is only used to a limited extent, a situation seen in several developing countries (Jørs et al. 2006; Maroni et al. 2005; Eddleston et al. 2002; Gomes et al. 1999). Other explanations of the limited use of personal protective equipment might be the costs of this equipment, and the inappropriateness for hot climates (Jørs, 2004).

A study from Denmark was not able to show any differences in chromosomal aberrations between pesticide applicators and controls, but showed that the genotoxic effects were linked to re-entry when workers handled newly sprayed crops (Lander et al. 2000). This was probably due to sufficient protective measures, a good hygiene during spraying operation, and the use of less toxic pesticides.

The cytogenetic alterations found among the farmers might mean that they have a higher risk of getting cancer (Møller, 2006a; Shadnia et al. 2005; Sigurdson et al. 2005; Hagmar et al. 2004), and various epidemiologic studies indicate that exposure to pesticides is associated with increased risk of cancer, especially leukemias, soft tissue sarcoma and prostate cancer (www.iarc.fr, Mills et al. 2001). At the individual cell level, however, the predictive value for cancer is still uncertain as no prospective cohort studies have been performed (Møller, 2006; Hagmar et al. 2004).

To prevent genetic damage in humans a better education of farmers and technicians, banning of the most toxic pesticides and promotion of ecological methods are possible options to improve the current serious situation in most developing countries (Jørs, 2004; Konradsen et al. 2003; Eddleston et al. 2002). To evaluate the risk of cancer further epidemiological studies in these populations are recommended.

## Figures and Tables

**Table 1:** The 10 most used pesticides among farmers in the area of investigation.

Active ingredient	WHO classification of toxicity	Generic class	Use
methamidophos	Ib	Organophosphate	Insecticide
sulphur	U		Fungicide/Insecticide
propenofos	II	Organophosphate	Insecticide
dimethoate	II	Organophosphate	Insecticide
cypermethrin	II	Pyrethroid	Insecticide
methyl parathion	Ia	Organophosphate	Insecticide
spinosad	U		Insecticide
propineb	U		Fungicide
permethrin	II	Pyrethroid	Insecticide
carbendazim	U		Fungicide

**Table 2:** Characteristics of the study group.

	Exposed (n = 48)	Controls (n = 33)
Age (mean ± SD)	37.77 ± 10.23	36.73 ± 9.50
Sex*		
Male	41	19
Female	7	14
Smoking		
Smokers	12	4
Non-smokers	36	29
Altitude, above sea level*		
<2500 m	21	22
>2500 m	27	11

* Significant differences between referent and exposed groups (p < 0.01)

**Table 3:** Percentage of lymphocytes with chromosome aberrations (CA) in pesticide exposed farmers and controls.

Exposed and controls	Living altitude	N	Chromosome breaks	Chromosome gaps	Chromatide breaks	Chromatide gaps	Total CA
			Mean ± SD	Mean ± SD	Mean ± SD	Mean ± SD	Mean ± SD
Controls		32	0.59 ± 1.73	0.22 ± 0.55	1.00 ± 2.27	0.72 ± 1.42	2.53 ± 4.79
	high	11	0.64 ± 2.11	0.18 ± 0.40	1.18 ± 2.18	0.45 ± 1.04	2.45 ± 5.11
	low	21	0.57 ± 1.57	0.24 ± 0.62	0.90 ± 2.36	0.86 ± 1.59	2.57 ± 4.75
0 applications past month		16	2.50 ± 3.79	0.75 ± 1.18	2.00 ± 2.63	0.94 ± 1.48	6.19 ± 7.96
	high	8	1.13 ± 3.18	0.50 ± 1.41	0.38 ± 1.06	0.63 ± 1.77	2.63 ± 7.42
	low	8	3.88 ± 4.05	1.00 ± 0.93	3.63 ± 2.77	1.25 ± 1.16	9.75 ± 7.19
1 application past month		12	2.25 ± 2.83	0.83 ± 1.75	2.00 ± 3.25	0.83 ± 1.34	5.92 ± 7.54
	high	5	1.60 ± 3.05	0.40 ± 0.89	0.80 ± 1.30	0.00 ± 0.00	2.80 ± 5.22
	low	7	2.71 ± 2.81	1.14 ± 2.19	2.86 ± 4.02	1.43 ± 1.51	8.14 ± 8.49
≥2 applications past month		19	4.21 ± 4.22	1.57 ± 1.80	4.53 ± 5.14	1.47 ± 2.01	11.79 ± 11.44
	high	14	5.21 ± 4.19	2.00 ± 1.88	5.71 ± 5.35	1.86 ± 2.18	14.79 ± 11.27
	low	5	1.40 ± 3.13	0.40 ± 0.89	1.20 ± 2.68	0.40 ± 0.89	3.40 ± 7.60

**Table 4:** GLM analysis on Poisson distribution of chromosome aberrations (CA) in pesticide exposed farmers and controls including sex and altitude in the model, figures show estimates and their 95% confidence intervals.

Exposed and controls	Chromosome breaks	Chromosome gaps	Chromatide breaks	Chromatide gaps	Total CA
Controls	0.61 (0.19 to 1.02)	0.28 (−0.05 to 0.61)	1.50 (0.83 to 2.18)	0.46 (0.09 to 0.83)	3.48 (2.66 to 4.31)
0 applications past month	2.01 (1.18 to 2.84)	0.68 (0.12 to 1.25)	1.52 (0.61 to 2.43)	0.46 (−0.03 to 0.95)	3.87 (2.60 to 5.13)
1 application past month	2.25 (1.34 to 3.15)	0.54 (0.03 to 1.05)	0.99 (0.18 to 1.80)	−0.22 (−1.05 to 0.61)	3.22 (1.85 to 4.60)
=2 applications past month	2.95 (2.08 to 3.82)	1.36 (0.70 to 2.03)	2.84 (1.73 to 3.95)	0.96 (0.31 to 1.61)	8.29 (6.54 to 10.03)
Sex female	−2.06 (−2.86 to −1.26)	−0.52 (−1.05 to 0.01)	−1.42 (−2.10 to −.73)	−1.01 (−1.62 to −0.39)	−3.32 (−4.13 to −2.51)
Altitude low	−0.01 (−0.50 to 0.49)	−0.02 (−0.38 to 0.34)	−0.29 (−1.10 to 0.51)	0.76 (0.11 to 1.42)	0.29 (−0.33 to 0.90)

**Table 5:** Comet assay parameters in lymphocytes of exposed farmers and controls.

Exposed and controls	Living altitude	N	Tail length	% DNA in the tail	Area of the tail	Tail moment
			
			Mean ± SD	Mean ± SD	Mean ± SD	Mean ± SD
Controls		32	124.12 ± 94.28	14.53 ± 9.67	28.73 ± 29.89	35.33 ± 42.95
	high	11	155.82 ± 87.74	17.32 ± 9.94	45.82 ± 34.00	37.91 ± 40.38
	low	21	108.27 ± 95.34	13.13 ± 9.45	20.18 ± 24.11	34.05 ± 45.05
0 applications past month		16	212.63 ± 112.27	23.85 ± 12.64	48.19 ± 42.89	63.25 ± 60.77
	high	8	234.63 ± 121.02	25.30 ± 12.47	70.13 ± 48.83	77.00 ± 77.68
	low	8	190.63 ± 106.10	22.41 ± 13.49	26.25 ± 21.39	49.50 ± 38.04
1 application past month		12	270.62 ± 126.58	31.71 ± 13.21	86.33 ± 59.29	106.38 ± 76.11
	high	5	287.80 ± 124.79	33.28 ± 16.32	79.60 ± 65.69	118.60 ± 75.61
	low	7	259.88 ± 135.00	30.72 ± 12.01	91.14 ± 59.18	98.75 ± 80.56
=2 applications past month		19	194.05 ± 112.11	23.76 ± 13.59	53.58 ± 43.74	63.32 ± 67.67
	high	14	227.21 ± 107.60	27.28 ± 13.77	65.07 ± 43.73	78.14 ± 72.87
	low	5	101.20 ± 65.61	13.88 ± 7.00	21.40 ± 25.27	21.80 ± 20.57

**Table 6:** GLM analysis after logarithmic transformation of comet assay parameters in pesticide exposed farmers and controls, including sex and altitude in the model, figures show the estimates and their 95% confidence intervals.

Exposed and controls	Tail length	% DNA in the tail	Area of the tail	Tail moment
Controls	5.04 (4.69 to 5.39)	2.85 (2.49 to 3.20)	3.79 (3.29 to 4.29)	3.75 (3.08 to 4.41)
0 applications past month	0.46 (0.07 to 0.85)	0.43 (0.04 to 0.83)	0.34 (−0.27 to 0.96)	0.57 (−0.20 to 1.34)
1 application past month	0.73 (0.37 to 1.10)	0.75 (0.40 to 1.10)	0.98 (0.45 to 1.51)	1.09 (0.45 to 1.74)
=2 applications past month	0.32 (−0.08 to 0.72)	0.40 (0.01 to 0.78)	0.34 (−0.23 to 0.91)	0.51 (−0.22 to 1.24)
Sex female	−0.06 (−0.38 to 0.26)	−0.03 (−0.35 to 0.28)	−0.44 (−1.01 to 0.12)	0.03 (−0.51 to 0.57)
Altitude low	−0.29 (−0.54 to −0.03)	−0.23 (−0.48 to 0.01)	−0.45 (−0.81 to −0.08)	−0.34 (−0.77 to 0.08)
